# Effect of Intermittent Fasting on Body Image Satisfaction and Appreciation Among Saudi Adults

**DOI:** 10.7759/cureus.33468

**Published:** 2023-01-06

**Authors:** Ghadeer Dairi, Renad Alafghani, Khloud Ghafouri, Essra Noorwali

**Affiliations:** 1 Department of Clinical Nutrition, Armed Forces Hospital, Taif, SAU; 2 Department of Clinical Nutrition, Faculty of Applied Medical Sciences, Umm Al-Qura University, Makkah, SAU

**Keywords:** body appreciation, body image satisfaction, saudi arabia, weight loss, intermittent fasting, obesity

## Abstract

Background: Obesity remains a public health concern, and intermittent fasting (IF) is a popular strategy for weight loss that has gained considerable scientific and popular attention. However, weight control can affect body image. Poor body image and its impact on psychological well-being are linked to obesity in many individuals. Body appreciation is a crucial characteristic of positive body image. However, there is a lack of studies assessing these relationships in Saudi adults.

Aim: To evaluate the associations between IF diet, body image satisfaction, and body appreciation in Saudi adults.

Method: A web-based cross-sectional study was done with healthy Saudi adults aged 18 to 50 years old who followed the IF diet. The questionnaire had five sections: a brief study introduction, sociodemographic variables, adherence to the diet, modified body image scale, and body appreciation scale. Ethical approval was obtained.

Results: A total of 190 participants were included in this study, including 59 (31.1%) males and 131 (68.9%) females. Comparing before and after IF, the body mass index (BMI) significantly decreased after IF (p < 0.001). After IF, a significant decrease in the body image dissatisfaction score was observed (p < 0.001). A significant increase was noted in the body appreciation score (p < 0.001).

Conclusion: IF is significantly associated with better body image satisfaction and body appreciation. These results may help weight loss studies and may have public health implications.

## Introduction

Obesity is a disease in which the body has excessive fat, resulting in impairment of physical and psychological health and well-being. It remains a significant public health concern and issue. It is mostly induced by the interaction of multiple factors, including environmental, genetic, nutritional, physical, and behavioral factors. It has been noted that obese people have a high risk for developing chronic diseases, type 2 diabetes, cardiovascular disease, and some cancers [1,2]. Therefore, studying factors associated with weight loss is highly needed.

Overweight and obesity prevalence are rapidly increasing worldwide. In 2016, more than 1.9 billion adults were overweight, including 650 million who were suffering from obesity, and approximately 2.8 million adults die each year due to the condition [3]. If the prevalence of obesity continues rising, by 2030, an estimated 38% of the globe's adult population will be overweight, and another 20% will be obese [2]. In the Kingdom of Saudi Arabia, large nationwide population-based surveys predicted a remarkable increase in obesity prevalence by 2022 [4]. Moreover, Saudi Arabia is now among the nations with the highest prevalence rates of obesity and overweight due to a number of contributing factors, including a sedentary lifestyle and a high-calorie diet [1].

Intermittent fasting (IF) is a popular strategy for weight loss, which may present independent health benefits. It has gained considerable scientific and popular attention and has been introduced as a feeding method under certain conditions in clinical practice [5]. It has many forms, but the basic premise involves taking periodic breaks from eating [6]. One common form of IF is fasting for up to 24 h once or twice a week with ad libitum food intake for the remaining days, which is known as periodic prolonged fasting or intermittent calorie restriction. Other forms are time-restricted feeding, such as eating for only eight hrs and then fasting for the other 16 hrs of the day, and alternate-day fasting (ADF). Most ADF programs involve alternating feast (ad libitum intake) and fast days (≤25% of energy needs), with some protocols allowing no caloric intake on fast days [7,8].

A huge body of evidence shows that body-weight control can affect body image. Body image is a subjective and multidimensional construct. It is composed of the perceptions (i.e., perceptual component) and the emotions, cognitions, and attitudes (i.e., evaluative component) that we have toward our body, which are translated into bodyweight and appearance-management efforts (i.e., behavioral component) [9]. However, weight misperception may limit the effectiveness of obesity prevention and management efforts [10]. Personal perceptions about the body refer to how a person considers their own body weight, size, shape, and composition to be. Poor body image and its impact on psychological well-being are inextricably linked to obesity in many individuals. Therefore, determining whether weight loss interventions affect body image in obese individuals is a worthwhile endeavor [11].

Body appreciation has been defined as accepting, holding favorable opinions toward, and respecting the body, while also rejecting media-promoted appearance ideals as the only form of human beauty. Studies have identified body appreciation as a key characteristic of positive body image, which is more narrowly described in these investigations as gratitude toward the body. Other positive body image characteristics detected in previous studies include body acceptance and love, inner positivity influencing outer demeanor, and a broad conceptualization of beauty. These appear to fit within the scope of the definition of body appreciation which is operationalized and measured via the 13-item body appreciation scale [12]. To our knowledge, this is the first study that evaluates the association between IF and body image satisfaction and appreciation. We hypothesize that people who fast for up to 16 hours per day develop a sense of accomplishment, which boosts self-esteem and thus body appreciation. To approach this hypothesis, we aimed to investigate the association between IF, body image satisfaction, and body appreciation among Saudi adults. The specific objectives are to compare body mass index (BMI), body image satisfaction, and appreciation before and after following the IF diet and to identify factors associated with body image satisfaction and body appreciation after following the IF diet.

## Materials and methods

Study design and participants

This study was a web-based cross-sectional survey. The questionnaire was distributed from January 2022 to February 2022. The sample for this study included participants aged 18 years and older who followed the IF diet with healthy medical records in Saudi Arabia. Participants received an online questionnaire through WhatsApp™, Twitter™, email, and other social media platforms.

Exclusion criteria

Participants were excluded from this study if they did not follow the IF diet, had a chronic health problem, were following a therapeutic diet, had severe psychological disorders, were pregnant, or were younger than 18 years old.

Ethical consideration

Our research was approved by the Biomedical Research Ethics Committee, Umm Al-Qura University (Approval No. HAPO-02K-012-2022-2-933).

Main outcome measures

The questionnaire was obtained from previous studies [12,13]. Google Forms was used to easily distribute it via social media platforms. The first section included a brief introduction of the study, the purpose of conducting the study, the time expected to complete the questionnaire, the researcher's e-mail address in case of any concerns or questions, and clarification indicating that the participant's information is confidential and that participation is anonymous. Before proceeding with the questionnaire, participants consented to the following statement: “I express my agreement to participate in this study by completing the questionnaire. Before finishing the questionnaire, I can freely and without consequences terminate my participation.” The second section of the questionnaire collected sociodemographic variables including gender, age, nationality, social status, place of residence, educational level, career, and health status. The third section contained self-reported anthropometric measurements and the duration of following the IF diet. The fourth and fifth sections included a body image scale that measures body image satisfaction [13] and the Body Appreciation Scale 2 (BAS-2) [12].

On the body image satisfaction scale, participants scored each question from 0 (“Not at all”) to 3 (“Very much”), and the possible score range was 0 to 27, with higher scores indicating higher body image dissatisfaction [13]. On the Body Appreciation Scale, participants scored each question from 1 (“never”) to 5 (“Always”). The possible score range was 1 to 50, with a higher score reflecting more body appreciation [12]. If the score is less than 50%, it was considered a low body image dissatisfaction level. If the score was 50-75%, it was indicated as a moderate body image dissatisfaction level, and higher than 75% showed a high body image dissatisfaction level.

Variables

BMI was calculated using the participants’ self-reported weights and heights in the questionnaire. Participants’ weights in kilograms were divided by their heights in meters squared (BMI = weight in kg/height^2^ {in m^2^}). We categorized the BMIs according to the guidelines of the World Health Organization (WHO): underweight - BMI below 18.5 kg/m^2^, normal weight - BMI greater than or equal to 18.5 to 24.9 kg/m^2^, pre-obesity - BMI greater than or equal to 25 to 29.9 kg/m^2^, obesity class I - BMI greater than or equal to 30 to 34.9 kg/m^2^, obesity class II - BMI greater than or equal to 35 to 39.9 kg/m^2^, obesity class III - BMI greater than 40 kg/m^2^ [3,14].

Survey translation

In order to translate the survey tool into Arabic, a forward-backward translation method was used. It was translated from English to Arabic by two researchers, revised by two different researchers, and approved by the Biomedical research ethics committee. To maintain the accuracy of the translated statements, we put more emphasis on conceptional translation than word-by-word translation.

Piloting study

The questionnaire tool was evaluated and validated by clinical nutritionists from Umm Al-Qura University. They were questioned regarding the questions' simplicity and understandability, as well as their validity on their face and whether any of them were challenging to comprehend. They were also questioned about whether any of the inquiries offended or upset them. They claimed the questionnaire was easy to comprehend and complete. Before the questionnaire was used on a broader scale, pilot research with a small group of participants was carried out to gauge comprehension, and they confirmed that it is simple and clear.

Statistical analysis

Data analysis was performed using the Statistical Package for the Social Sciences (SPSS) version 23 (IBM Corp., Armonk, NY). The frequency and percentages were used to display categorical variables. The mean and standard deviation were used to display continuous variables. Paired t-tests were used to determine if there was a significant change in the BMI, body appreciation score, and body image dissatisfaction after following an IF diet compared to before following the diet. An independent t-test and ANOVA test were also used to determine factors associated with the body image dissatisfaction score and body appreciation score. An ANOVA test followed by Tukey’s post-hoc test was used to determine differences between groups. Pearson’s correlation coefficient was used to determine the correlation between the two variables. A coefficient < 1 means that there is a strong negative correlation, while a coefficient > 1 means that there is a strong positive correlation. The level of significance was set at 0.05.

## Results

Participants' socio-demographic profiles

Out of 432 responders, a total of 190 participants met our study criteria. Table 1 shows the socio-demographic profile of the participants. The mean age of participants was 31.93 + 8.63 years, 59 (31.1%) were males, and 131 (68.9%) were females. 188 (98.9%) were Saudis, while only 2 (1.1%) were non-Saudi. Seventy-eight (41.1%) were single, 111 (58.4%) were married, and 1 (0.5%) was divorced/widowed. Regarding the place of residence, 9 (4.7%) were from the northern region of the country, 9 (4.7%) were from the southern region, 28 (14.7%) were from the central region, 12 (6.3%) were from the eastern region, and 132 (69.5%) were from the western region. Regarding education, 1 (0.5%) had a primary school education, 16 (8.4%) had a high school education, 16 (8.4%) had a diploma, 117 (61.6%) had a bachelor’s degree, and 40 (21.1%) had a higher education (masters/PhD). Regarding employment, 23 (12.1%) were students, 102 (53.7%) were employed, 4 (2.1%) were retired, and 61 (32.1%) were not working. Nine (4.7%) participants reported using medications, 37 (19.5%) reported using supplements, 4 (2.1%) reported using both medications and supplements, and 140 (73.7%) reported using none of these.

**Table 1:** Demographic characteristics of the study participants

Demographical Characteristics	Frequency	%
Mean age	31.93 years	
Standard deviation	8.63 years	
Gender		
Male	59	31.10
Female	131	68.90
Nationality		
Saudi	188	98.90
Non-Saudi	2	1.10
Marital Status		
Single	78	41.10
Married	111	58.40
Divorced/widowed	1	0.50
Place of Residency		
Northern region	9	4.70
Southern region	9	4.70
Central region	28	14.70
Eastern region	12	6.30
Western region	132	69.50
Education		
Primary school	1	0.50
High school	16	8.40
Diploma	16	8.40
Bachelor's degree	117	61.60
Higher education (masters/PhD)	40	21.10
Employment		
Student	23	12.10
Employed	102	53.70
Retired	4	2.10
Not working	61	32.10

Participants' BMI before and after following the IF diet

As illustrated in Table 2, the mean BMI before the IF diet was 31.23 + 5.88, while after following the diet, the mean BMI was 26.75 + 4.77. Only 12 (6.3%) participants followed the diet for less than 1 month, while 17 (8.9%) participants followed the diet for 1 month. Thirty-one (16.3%) participants followed the diet for two months, and 48 (25.3%) participants followed the diet for three months. The majority of the responders (82 participants; 43.2%) followed the diet for four months or more. The mean number of fasting hours during the IF diet was 15.49 + 2.35 hours.

**Table 2:** Intermittent fasting diet profile

Question	Frequency	%
BMI before the Intermittent Fasting Diet		
Mean	31.23	
Standard deviation	5.88	
BMI after the Intermittent Fasting Diet		
Mean	26.75	
Standard deviation	4.77	
For how long have you been following the intermittent fasting diet?		
Less than one month	12	6.3
One month	17	8.9
Two months	31	16.3
Three months	48	25.3
Four months or more	82	43.2
Fasting Hours During the Intermittent Fasting Diet		
Mean	15.49 hours	
Standard deviation	2.35 hours	

None of the participants before the IF diet were underweight. However, only two (1.1%) of participants were underweight after following the IF diet. Furthermore, 23 (12.1%) showed a normal weight before the IF diet compared to 78 (41.1%) after following the diet. Sixty-five (31.6%) participants were overweight before the diet compared to 59 (31.1%) participants after the diet. One hundred and seven (56.3%) participants were obese before the diet, which decreased to 51 (26.8%) after following the diet.

Body image dissatisfaction score before and after following the IF diet

Participants’ body image dissatisfaction decreased after the IF diet compared to before the diet. As illustrated in Table 3, the mean body image dissatisfaction score before the diet was 15.25 + 7.07, while after following the diet, it decreased to 8.17 + 6.9 (Table 4). Around 26.3% of the participants had a moderate body image dissatisfaction level before the diet, while 10% had a moderate body image dissatisfaction level after following the diet. 29.5% had a high body image dissatisfaction level before following the diet compared to 5.8% after following the diet.

**Table 3:** Participants’ body image dissatisfaction before and after the intermittent fasting diet

Question	Before the Intermittent Fasting Diet		After the Intermittent Fasting Diet	
	Frequency	%	Frequency	%
Have you been feeling self-conscious about your appearance?				
Not at all	35	18.40	91	47.90
A little	38	20.00	53	27.90
Quite a bit	56	29.50	39	20.50
Very much	61	32.10	7	3.70
Have you felt less physically attractive as a result of your disease or treatment?				
Not at all	37	19.50	97	51.10
A little	39	20.50	53	27.90
Quite a bit	52	27.40	29	15.30
Very much	62	32.60	11	5.80
Have you been dissatisfied with your appearance when you dressed?				
Not at all	24	12.60	88	46.30
A little	37	19.50	65	34.20
Quite a bit	61	32.10	25	13.20
Very much	68	35.80	12	6.30
Have you been feeling less feminine/masculine as a result of your disease or treatment?				
Not at all	59	31.10	106	55.80
A little	30	15.80	50	26.30
Quite a bit	47	24.70	19	10.00
Very much	54	28.40	15	7.90
Do you find it difficult to look at yourself naked?				
Not at all	53	27.90	98	51.60
A little	36	18.90	50	26.30
Quite a bit	48	25.30	28	14.70
Very much	53	27.90	14	7.40
Have you been feeling less sexually attractive as a result of your disease or treatment?				
Not at all	49	25.80	102	53.70
A little	33	17.40	51	26.80
Quite a bit	52	27.40	23	12.10
Very much	56	29.50	14	7.40
Do you avoid people because of the way you feel about your appearance?				
Not at all	63	33.20	111	58.40
A little	34	17.90	48	25.30
Quite a bit	48	25.30	21	11.10
Very much	45	23.70	10	5.30
Do you feel the disease or treatment has left your body less whole?				
Not at all	26	13.70	79	41.60
A little	33	17.40	55	28.90
Quite a bit	52	27.40	37	19.50
Very much	79	41.60	19	10.00
Have you felt dissatisfied with your body?				
Not at all	29	15.30	81	42.60
A little	37	19.50	61	32.10
Quite a bit	57	30.00	34	17.90
Very much	67	35.30	14	7.40

**Table 4:** Body image dissatisfaction score

Measure	Before the Intermittent Fasting Diet	After the Intermittent Fasting Diet
Mean	15.25	8.17
Standard deviation	7.07	6.9

Body appreciation assessment before and after following the IF diet

Table 5 displays the participants’ body appreciation before and after the IF diet. The mean body appreciation score before the IF diet was 31.59 + 11.34, while after following the diet, it increased to 39.68 + 10.36 (Table 6). Scores less than 50% were considered low body appreciation levels, 50-75% were considered a moderate body appreciation level, and higher than 75% was considered a high body appreciation level. Out of the total, 36.8% of the participants had a moderate body appreciation level before the diet, while 20.5% had a moderate body appreciation level after following the diet, 35.8% of the participants had a high body appreciation level before following the diet compared to 67.9% after following the diet.

**Table 5:** Assessment of participants' body appreciation before and after the intermittent fasting diet

Question	Before the Intermittent Fasting Diet		After the Intermittent Fasting Diet	
	n	%	n	%
I respect my body.				
Never	25	13.20	6	3.20
Seldom	27	14.20	21	11.10
Sometimes	46	24.20	19	10.00
Often	46	24.20	49	25.80
Always	46	24.20	95	50.00
I feel good about my body.				
Never	33	17.40	12	6.30
Seldom	23	12.10	13	6.80
Sometimes	60	31.60	31	16.30
Often	40	21.10	52	27.40
Always	34	17.90	82	43.20
I feel that my body has at least some good qualities.				
Never	29	15.30	15	7.90
Seldom	22	11.60	14	7.40
Sometimes	57	30.00	24	12.60
Often	39	20.50	55	28.90
Always	43	22.60	82	43.20
I take a positive attitude towards my body.				
Never	26	13.70	4	2.10
Seldom	32	16.80	18	9.50
Sometimes	56	29.50	24	12.60
Often	36	18.90	52	27.40
Always	40	21.10	92	48.40
I am attentive to my body’s needs.				
Never	28	14.70	7	3.70
Seldom	38	20.00	18	9.50
Sometimes	54	28.40	34	17.90
Often	38	20.00	54	28.40
Always	32	16.80	77	40.50
I feel love for my body.				
Never	28	14.70	9	4.70
Seldom	25	13.20	12	6.30
Sometimes	52	27.40	28	14.70
Often	46	24.20	53	27.90
Always	39	20.50	88	46.30
I appreciate the different and unique characteristics of my body.				
Never	29	15.30	15	7.90
Seldom	24	12.60	17	8.90
Sometimes	46	24.20	32	16.80
Often	47	24.70	43	22.60
Always	44	23.20	83	43.70
My behavior reveals my positive attitude toward my body; for example, I walk holding my head high and smiling.				
Never	26	13.70	11	5.80
Seldom	41	21.60	15	7.90
Sometimes	50	26.30	30	15.80
Often	37	19.50	51	26.80
Always	36	18.90	83	43.70
I am comfortable in my body.				
Never	37	19.50	12	6.30
Seldom	32	16.80	14	7.40
Sometimes	56	29.50	29	15.30
Often	35	18.40	52	27.40
Always	30	15.80	83	43.70
I feel like I am beautiful even if I am different from media images of attractive people (e.g., models, actresses/actors).				
Never	28	14.70	15	7.90
Seldom	39	20.50	19	10.00
Sometimes	39	20.50	25	13.20
Often	36	18.90	47	24.70
Always	48	25.30	84	44.20

**Table 6:** Body appreciation score

Measure	Before the Intermittent Fasting Diet	After the Intermittent Fasting Diet
Mean	31.59	39.68
Standard deviation	11.34	10.36

Weight and BMI significantly decreased after the diet

Table 7 shows that weight and BMI significantly decreased after the IF diet compared to before. The mean BMI for participants before the IF diet was 31.23 ± 5.88 compared to 26.75 + 4.77 after the diet (p < 0.001). A significant decrease in the body image dissatisfaction score was observed (p < 0.001) when comparing the before-diet score and the after-diet score (15.25 + 7.07 before the diet vs. 8.17 + 6.9 after the diet). A significant increase was also noted in the body appreciation score (p < 0.001) when comparing the before-diet score and after-diet score (31.59 + 11.34 before the diet vs. 39.68 + 10.36 after the score) (p<0.001).

**Table 7:** Comparison of participants’ BMI, body image dissatisfaction, and body appreciation before and after intermittent fasting diet *Significant at level 0.05 Note: Paired T-test was used for these variables.

Variable	BMI Before Intermittent Fasting Diet	BMI After Intermittent Fasting Diet	P-Value
BMI (Mean (standard deviation)	31.23 (5.88)	26.75 (4.77)	< 0.001*
Body Image Dissatisfaction Score (Mean (standard deviation)	15.25 (8.17)	7.07 (6.90)	< 0.001*
Body Appreciation Score (Mean (standard deviation)	31.59 (11.33)	39.65 (10.36)	< 0.001*

Table 8 shows the factors associated with the body image dissatisfaction score measured after the IF diet. A significant difference was observed in the body image dissatisfaction score measured after the IF diet between males and females (p = 0.049): males had a significantly higher body image dissatisfaction score than females (8.54 + 7.58 vs. 6.41 + 6.49). The use of medication/supplements was also significantly associated with the body image dissatisfaction score measured after the IF diet (p = 0.032). The Tukey post-hoc test revealed that those taking a supplement had a significantly higher dissatisfaction score than those who did not use medication or supplements (p < 0.05). The number of fasting hours showed a significant, weak, positive correlation with the body image dissatisfaction score measured after the IF diet (p = 0.003, correlation coefficient = 0.216) (Table 9). Marital status, place of residence, education, employment, duration of following the IF diet, and age were all not significantly associated with the body image dissatisfaction score measured after the IF diet.

**Table 8:** Factors associated with body image dissatisfaction score after following the intermittent fasting diet *Significant at level 0.05

Factor	Body Image Dissatisfaction Score After Following the Intermittent Fasting Diet		P-Value
	Mean	Standard deviation	
Gender			0.049*
Male	8.54	7.58	
Female	6.41	6.49	
Marital Status			0.202
Single	6.28	6.29	
Married	7.59	7.29	
Place of Residency			0.073
Northern region	6.33	6.82	
Southern region	5.11	5.80	
Central region	10.50	8.92	
Eastern region	6.33	7.74	
Western region	6.60	6.26	
Education			0.483
High school	6.69	6.64	
Diploma	7.25	7.12	
Bachelor's degree	6.64	6.73	
Higher education (master/PhD)	8.60	7.41	
Employment			0.615
Student	6.30	5.83	
Employed	7.56	7.00	
Not working	6.69	7.08	
The use of medications or supplements following the intermittent fasting diet			0.032*
Medications	8.44	6.98	
Supplements	9.70	7.42	
None	6.44	6.66	
The duration of following the intermittent fasting diet			0.440
Less than one month	10.00	9.28	
One month	6.24	6.80	
Two months	7.03	7.50	
Three months	6.02	6.83	
Four months or more	7.45	6.33	

**Table 9:** Correlation between the age and body image dissatisfaction score after following the intermittent fasting diet *Significant at level 0.05

Correlation Between the Age and Dissatisfaction Score	
P-value	0.117
Correlation coefficient	0.108
Correlation between Number of Fasting hours and Body Image Dissatisfaction Score	
P-value	0.003*
Correlation coefficient	0.216

Factors associated with body appreciation score

Tukey post-hoc test revealed that those taking supplements had a significantly lower appreciation score when compared to those who did not use medication or supplements (p < 0.05). Gender, marital status, place of residency, education, employment, duration of following the IF diet, age, and number of fasting hours were all not significantly associated with the body appreciation score measured after the IF diet (Table 10). Table 11 shows the factors correlated with the body appreciation score measured after the IF diet. The use of medication/supplements was significantly associated with the body appreciation score measured after the IF diet (p = 0.029).

**Table 10:** Factors associated with body appreciation score after following the intermittent fasting diet *Significant at level 0.05

Factor	Body Appreciation Score After Following the Intermittent Fasting Diet		P-Value
	Mean	Standard deviation	
Gender			0.974
Male	39.61	10.43	
Female	39.66	10.38	
Marital Status			0.673
Single	40.06	9.55	
Married	39.41	10.96	
Place of Residency			0.912
Northern region	37.89	10.41	
Southern region	42.00	9.11	
Central region	40.07	10.04	
Eastern region	40.83	9.53	
Western region	39.41	10.68	
Education			0.550
High school	43.06	8.89	
Diploma	38.50	10.26	
Bachelor's degree	39.48	10.42	
Higher education (masters/PhD)	38.98	10.85	
Employment			0.408
Student	41.57	7.95	
Employed	39.89	9.78	
Not working	38.33	12.17	
The use medications or supplements following the intermittent fasting diet			0.029*
Medications	37.67	12.28	
Supplements	35.73	10.52	
None	40.70	10.01	
The duration of following the intermittent fasting diet			0.936
Less than one month	40.83	11.63	
One month	38.00	11.59	
Two months	39.32	11.34	
Three months	39.33	10.46	
Four months or more	40.12	9.67	

**Table 11:** Correlation of age and number of fasting hours with body appreciation score after following the intermittent fasting diet *Significant at level 0.05

Correlation between the Number of Fasting hours and Body Appreciation Score	
P-value	0.208
Correlation coefficient	-0.092
Correlation between the Number of Fasting hours and Body Appreciation Score	
P-value	0.781
Correlation coefficient	-0.02

## Discussion

The prevalence of obesity is rising worldwide along with the subsequent health-related issues that it causes in both physical aspects (e.g., diabetes mellitus type II and hypertension) and psychosocial aspects (e.g., body image dissatisfaction and poor body appreciation). Thus, losing and maintaining a healthy weight has been a major concern for people in all age groups [3,12,15]. Fasting has recently been a trending method for safe weight loss [16-18]. The aim of this study was to evaluate the effect of IF on body image dissatisfaction and appreciation among Saudi adults.

We found that following the IF diet for less than one month to more than four months resulted in a significant drop in weight (from a mean of 83.2 kg to 71.2 kg) and BMI (from a mean of 31.2 to 26.75). These results are consistent with the study by Welton et al., who systematically reviewed 27 trials of IF and found that there was a loss of weight occurred among those who fasted in an intermittent manner, which ranged from 0.8% to 13% of baseline, as well as an average decline in BMI by 4.3% [19]. Collectively, these findings indicate that abstinence from food for a set period of time in a daily manner is an effective strategy for weight loss in the short term. These results paved the way for assessing whether weight loss through IF in particular has a positive impact on body image satisfaction and body appreciation.

In our study, the mean score of body image dissatisfaction was found to be 15.25 before following the IF diet and then decreased to 8.17 after following the IF diet. This reduction showed a significant positive correlation between IF and body image dissatisfaction (p < 0.001). This association might be related to the fact that the thinner someone is, the more his or her facial features become evident and detailed, thus increasing the attractiveness perceived by oneself and others. This can be reflected in the form of self-confidence, social praise, compliments, and encouragement, eventually increasing one’s sense of satisfaction with one’s body image.

An additional factor that should be taken into consideration is the psychosexual nature of men and women, who respectively tend to favor leanness and muscularity [20]. Both of these characteristics might be obscured to some degree by excessive fat but are regained with IF when weight is lost. Finally, nowadays, people are engaged in different social media platforms, where they may find themselves either consciously or unconsciously driven to compare their appearances with those of their peers, leading to body image dissatisfaction [21]. They subsequently try to look as similar as possible to their idealized figure, such as by losing weight by fasting intermittently, which ultimately leads to better body image satisfaction.

We found that the participants scored an average of 31.95 on BAS-2 before going through the IF diet, but it increased to 39.68 after following the IF diet, which may have been due to the participants’ weight loss. This increase showed a significant positive correlation between IF and body appreciation (p < 0.001). This association may be related to the consequences that being overweight or obese may impose on the body, which may improve with IF, leading to a better quality of life and body appreciation. Self-esteem and subjective perception of health have been identified as psychological factors affecting body appreciation [22-26].

We found that participants fasted for an average of 15.49 hours. In another study involving abdominally obese participants, the researchers found that 16 hours of fasting improved participants’ weight and waist circumference [27], which boosts self-esteem and therefore body appreciation. Although not measured in this study, participants probably made a decision about taking care of their bodies and health through engaging in health-promoting behaviors like physical activity and balanced nutrition, which enhance how health is perceived from a subjective point of view, eventually increasing body appreciation and decreasing body dissatisfaction.

Male gender has been found to significantly increase the score of dissatisfaction with body image in this study. These findings oppose the results of a study that found body image dissatisfaction to be significantly associated with the female gender [13,26,28-30]. In a previous study, males being more satisfied with their body image was explained by their tendency to pair the perfection of the body with functionality rather than appearance [31]. Furthermore, we assume that when males are overweight or obese, their fat is accumulated in the abdomen, giving them the appearance of an apple. This shape is cosmetically less appealing than the pear-shaped obesity of their female peers, making them more dissatisfied with their body image.

Participants taking supplements were observed to have a significantly higher score of body image dissatisfaction and a lower score of body appreciation compared to those who did not take supplements or medications. Likely, this is because people who are dissatisfied with their body image and do not appreciate it tend to take supplements such as multivitamins or minerals, believing that they could somehow have a positive effect on their body either functionally or cosmetically. Our result is consistent with several studies that found people taking dietary supplements tend to follow many healthy habits such as healthier nutritional practices and exercising regularly in order to maintain a healthy weight [16]. Moreover, many studies have found that people take supplements because they want to maintain their overall health, fulfill their nutritional needs, improve their physical appearance, and boost weight loss [16].

Marital status, place of residence, education, employment, and age were not found to have a significant association with body image dissatisfaction and body appreciation in our study. However, previous studies found that body image dissatisfaction does not change with age in women, and it is still unclear whether the level of body image dissatisfaction changes with age in men. Although body image dissatisfaction remains the same with age among women, appearance value tends to decline with age [32].

To our knowledge, this is the first study that assessed the relationship between IF, body image satisfaction, and body appreciation in a sample Saudi population. Furthermore, both genders were included in the study with ages ranging between 18 and 71 years. On the other hand, our study has some limitations. First, the survey was self-administered. This might have affected the accuracy of the results. Secondly, most of our participants were female (68.9%), so the results could be biased. One of the main limitations of an online survey is that some of the intended groups may have been missed, which may have affected how generalizable our results were. Finally, our sample size was too small to generalize our results to the Saudi population.

We recommend studying the effect of IF on body image satisfaction and body appreciation with data collected from males and females equally in a larger sample size so that less biased results can be obtained. We also recommend that future researchers examine the effects of IF on body image satisfaction and body appreciation in a group of people who are similar in age, education level, employment, and circumstances so that more accurate results can be obtained. Our findings could give insight to public health providers, especially clinical dietitians in Saudi Arabia. The results could help them to focus extensively on integrating IF practice regardless of the number of fasting hours. According to our preliminary results, this integration may improve body appreciation and lower dissatisfaction, which eventually might have an impact on not only the physical appearance of obese people but also their mental and emotional health. This study may be useful in raising the Saudi public’s health satisfaction and encouraging weight loss.

## Conclusions

In conclusion, IF was significantly associated with better body image satisfaction and body appreciation. Males had a significantly higher body image dissatisfaction compared to females, and the use of medication/supplements was an important factor correlated with body image dissatisfaction. These results may have significant implications for future studies using IF as a weight loss method. Further cohort studies are warranted to identify other variables influencing body image satisfaction and body appreciation. This will help in designing interventions that enhance patients’ satisfaction.
